# Tricuspid Valve in Congenital Heart Disease Patients: A Triad of Problems and No Remedy in Sight

**DOI:** 10.1016/j.jscai.2025.103805

**Published:** 2025-06-18

**Authors:** Zahid Amin, Niall Linnane

**Affiliations:** a Adult and Congenital Heart Disease, Advent Hospital for Children, Orlando, Florida; b Division of Cardiology, Texas Children’s Hospital, Baylor College of Medicine, Houston, Texas

**Keywords:** congenital heart disease, percutaneous, tricuspid valve

Tricuspid valve (TV) disease in combination with mitral valve disease is the most common valve disease in the United States. In patients without congenital heart disease (CHD), it is estimated that nearly 2 million people have clinically relevant tricuspid regurgitation (TR).^1^ The mortality rate is >45% when the TR progresses from moderate to severe.^2^ Thus, it has been demonstrated that TV disease is no longer a benign lesion and must be addressed when TR progresses.

Unfortunately, there is a lack of such data in patients with CHD. In patients without CHD, TR is usually secondary to either left ventricular dysfunction or left-sided valvular heart disease; sometimes it also follows atrial fibrillation that causes annular dilation. In patients with CHD, it occurs primarily because of dysplasia (Ebsteinoid and non-Ebsteinoid)^3^ in addition to being caused by right ventricular volume overload and/or pulmonary insufficiency with secondary annular dilation. Therefore, studying this heterogenous group of patients is difficult, and developing consensus guidelines is even more so. While surgical outcomes for TV repair in patients with Ebstein anomaly are excellent,^4^ there remains a gap when it comes to outcomes for repairing TV for other congenital conditions such as atrioventricular (AV) septal defects, pulmonary atresia, tetralogy of Fallot, and iatrogenic TR after ventricular septal defect closure with or without device.^5^

There are several reasons why the TV has been the least favorite valve for interventionalists and surgeons to address. The TV architecture is complex and not uniform in its anatomy, even in patients without CHD. While the TV typically contains 3 leaflets, recent work by Hahn et al^6^ has demonstrated the heterogeneity of the TV, and new nomenclature has been devised to assist planning of TV interventions. They describe 4 types, with only type I having the classic 3–leaflet anatomy. Only 54% had a type I classification. This variability makes planning for interventions extremely challenging for both surgeons and interventionalists. In addition to leaflet variability, the annulus of the valve is nonplanar, with elliptical shape. The posterior septal leaflet is the lowest (most ventricular), and the anteroseptal leaflet is the highest (most atrial). The leaflets are supported by fibrous chordae tendineae, papillary muscles, and right atrial and ventricular myocardium,^7^^,^^8^ all of which could become dysfunctional and require treatment. Finally, multiple structures interact intimately with the TV and must be considered during any intervention. Just below the anteroseptal commissure is the conduction tissue (AV node and bundle of His), which increases the risk of complete heart block during interventions. The right coronary artery is in the AV groove, and the coronary sinus lies adjacent to the posteroseptal commissure.^9^

The second reason the TV was ignored is primarily historic. The survival of patients with 1 functioning ventricle led to an article being written by stalwarts of surgery titled “The dispensable right ventricle.”^10^

The third reason TV has been ignored is because TR is usually secondary to left-sided valvular heart disease and annular dilation due to atrial fibrillation. In our quest to treat those issues, TV pathology was forgotten until recently; however, in all TV clinical trials currently registered on ClinicalTrials.gov investigating various transcatheter TV treatments (23 studies), patients with CHD and pulmonary hypertension (PHT) are excluded. Hence, we continue to have less understanding about this valve in the setting of CHD and PHT.

In this issue of *JSCAI*, Vera-Sarmiento et al^11^ presented a case series of different TV technologies (INTREPID [Medtronic] and TricValve [Products & Features]) used to lower the burden of TR in patients with polyvalvular disease and PHT. The series brings up fascinating discussion points about trying to introduce novel transcatheter therapies in “one-off” patients. We believe this is a positive step toward providing treatment to a very difficult subset of patients. Although the valve techniques used were not perfect, this is a definite step forward and aligns with transcatheter TV repair and valve-in-valve case reports published in patients with CHD.^12^^,^^13^ We believe that congenial and structural interventional colleagues can truly collaborate to help advance and use TV technologies in patients with CHD. Additionally, we believe that it is time to include patients with CHD and TR in clinical trials or we need to start trials on patient with CHD.

The authors should be commended for crossing the barriers to improve the quality of life of their patients with off-label use of these technologies. We hope to see collaboration between CHD and structural interventionalists to speak for this vulnerable population so patients with CHD can be included in future trials that will help establish future guidelines.
